# Identification of Prognostic Genes in the Tumor Microenvironment of Hepatocellular Carcinoma

**DOI:** 10.3389/fimmu.2021.653836

**Published:** 2021-04-07

**Authors:** Shixin Xiang, Jing Li, Jing Shen, Yueshui Zhao, Xu Wu, Mingxing Li, Xiao Yang, Parham Jabbarzadeh Kaboli, Fukuan Du, Yuan Zheng, Qinglian Wen, Chi Hin Cho, Tao Yi, Zhangang Xiao

**Affiliations:** ^1^Laboratory of Molecular Pharmacology, Department of Pharmacology, School of Pharmacy, Southwest Medical University, Luzhou, China; ^2^South Sichuan Institute of Translational Medicine, Luzhou, China; ^3^Department of Oncology and Hematology, Hospital (T.C.M) Affiliated to Southwest Medical University, Luzhou, China; ^4^Neijiang Health and Health Vocational College, Neijiang, China; ^5^Department of Oncology, Affiliated Hospital of Southwest Medical University, Luzhou, China; ^6^Faculty of Medicine, School of Biomedical Sciences, The Chinese University of Hong Kong, Hong Kong, China; ^7^School of Chinese Medicine, Hong Kong Baptist University, Hong Kong, China; ^8^Department of Pharmacy, The Affiliated Hospital of Southwest Medical University, Luzhou, China

**Keywords:** tumor microenvironment, Hepatocellular carcinoma, TCGA, ESTIMATE algorithm, Prognosis

## Abstract

**Background:** Hepatocellular carcinoma (HCC) is one of the most common malignant tumors in the world. The efficacy of immunotherapy usually depends on the interaction of immunomodulation in the tumor microenvironment (TME). This study aimed to explore the potential stromal-immune score-based prognostic genes related to immunotherapy in HCC through bioinformatics analysis.

**Methods:** ESTIMATE algorithm was applied to calculate the immune/stromal/Estimate scores and tumor purity of HCC using the Cancer Genome Atlas (TCGA) transcriptome data. Functional enrichment analysis of differentially expressed genes (DEGs) was analyzed by the Database for Annotation, Visualization, and Integrated Discovery database (DAVID). Univariate and multivariate Cox regression analysis and least absolute shrinkage and selection operator (LASSO) regression analysis were performed for prognostic gene screening. The expression and prognostic value of these genes were further verified by KM-plotter database and the Human Protein Atlas (HPA) database. The correlation of the selected genes and the immune cell infiltration were analyzed by single sample gene set enrichment analysis (ssGSEA) algorithm and Tumor Immune Estimation Resource (TIMER).

**Results:** Data analysis revealed that higher immune/stromal/Estimate scores were significantly associated with better survival benefits in HCC within 7 years, while the tumor purity showed a reverse trend. DEGs based on both immune and stromal scores primarily affected the cytokine–cytokine receptor interaction signaling pathway. Among the DEGs, three genes (CASKIN1, EMR3, and GBP5) were found most significantly associated with survival. Moreover, the expression levels of CASKIN1, EMR3, and GBP5 genes were significantly correlated with immune/stromal/Estimate scores or tumor purity and multiple immune cell infiltration. Among them, GBP5 genes were highly related to immune infiltration.

**Conclusion:** This study identified three key genes which were related to the TME and had prognostic significance in HCC, which may be promising markers for predicting immunotherapy outcomes.

## Introduction

Hepatocellular carcinoma (HCC), one of the digestive tract cancers, is also the most common primary liver cancer (1). Hepatocarcinogenesis is a multistep and complex biological process in which many signaling cascades are altered, resulting in heterogeneous molecular profiles and ultimately in tumorigenesis, progression, and metastasis (2). Surgical treatment and chemotherapy are the main therapies for HCC (3), but incidence rates of HCC are continuing to grow, and the probability is rising faster than any other cancer in both men and women (4). A systematic analysis for the Global Burden of Disease Study (GBD) has shown that the incidence and mortality of HCC rank among the top 10 cancers, and death in adults with cirrhosis is the leading cause for the mortality of HCC (5). With the improvement of medical standards, the treatment of HCC has indeed made progress. But currently, HCC treatment is still a global research hotspot, and more and more attention has been paid to cancer immunotherapy, one of the most promising methods for cancer treatment (6). Besides, studies have shown that immune tolerance and escape in the immunosuppressive microenvironment of HCC can be promoted by multiple mechanisms (7, 8). Therefore, it is essential to understand the microenvironment of HCC.

The tumor microenvironment (TME), which is comprised of a mixture of immune cells, stromal cells, cancer cells, the intricate cytokine and chemokines environment, and other components (6, 9), is a dynamic system. Immune cells and stromal cells within the TME are the two main types of non-tumor components which are considered to play important role in the diagnosis and prognosis of tumors (10). Evidence from studies indicates that stromal cells within the TME are genetically stable and are attractive therapeutic targets with reduced risk of resistance and tumor recurrence (11). In addition, due to the dysregulation of the metabolic activity of tumor cells, tumor-infiltrating immune cells usually experience metabolic stress, resulting in an impaired anti-tumor immune response (12). A multi-target approach that simultaneously suppresses TME components may provide a more effective method of treating cancer (13). Therefore, understanding the TME is critical for inhibiting tumorigenesis, invasion, and metastasis, and in effectively managing the immune response (14–16).

Bioinformatics resolve the problems of biology through the methods of applied mathematics, informatics, statistics, and computer science (17, 18). At the same time, with the growth of the amount of biological tumor data, bioinformatics is essential for the storage, analysis, and visualization of cancer immunotherapy data (19, 20). Its rapid development has provided a user-friendly and convenient platform for researchers, guiding the implementation of basic experiments (21, 22). In 2013, Yoshihara et al. created a method to infer the ratio of stromal cells and immune cells in malignant tumors through gene expression signatures that can be derived from The Cancer Genome Atlas (TCGA)–ESTIMATE algorithm. In addition, the algorithm can also predict tumor purity, which helps understand the influence of the microenvironment on neoplastic cells (23). Researchers have extensively verified and confirmed the accuracy of the prediction. In recent years, the ESTIMATE algorithm has been applied to glioblastoma (10), gastric cancer (16), clear cell renal cell carcinoma (9), colon cancer (24), and so on. However, the application of HCC remains to be elucidated.

In this study, we obtained transcriptome data of HCC from the TCGA database, and analyzed the immune/stromal/Estimate scores and tumor purity within the microenvironment using the ESTIMATE algorithm. The relationship between immune/stromal/Estimate scores and tumor purity with survival and clinical parameters were explored. Hub genes associated with immune and stromal scores and with prognostic values were chosen.

## Materials and Methods

### Data Processing

RNA sequencing (RNA-Seq) and clinicopathological data of patients with HCC were downloaded from Genomic Data Commons

(GDC) database (https://portal.gdc.cancer.gov/) (25, 26). About 374 tumor samples were used for the analysis. ESTIMATE algorithm was used to calculate the stromal/immune/Estimate scores and tumor purity using “estimate” package through the R project (http://r-forge.r-project.org; repos=rforge, dependencies=TRUE) (23). The Human Protein Atlas (HPA) (https://www.proteinatlas.org/about/download) was used to verify the immunohistochemistry staining of genes. Kaplan–Meier plotter (http://kmplot.com/analysis/) was applied to assess the prognostic value of the biomarkers.

### Differential Expression Analysis

The median was set as the cut-off value of the immune and stromal scores. Patient samples were divided into two groups, respectively, namely the high-score group and the low-score group. Differential expression analysis was analyzed on the matrix of the sample using the R package, LIMMA. The filtering conditions for the differential genes were as follows: Fold Change > | ±1|, with adjusted *p* < 0.05.

### Enrichment Analysis

The differentially expressed genes (DEGs) from differential expression analysis that meet the conditions were used for enrichment analysis, including Kyoto Encyclopedia of Genes and Genomes (KEGG) and Gene Ontology (GO) enrichment analysis *via* Database for Annotation, Visualization, and Integrated Discovery database (DAVID) function annotation tool (https://david.ncifcrf.gov) (27). GO contains biological processes (BPs), cell components (CCs), and molecular functions (MFs), and the signaling pathways were identified by considering both *p*-value and count number.

### Prognostic Gene Selection by Cox Regression Analysis and Least Absolute Shrinkage and Selection Operator (LASSO)

In order to select the survival-related genes, univariate Cox regression analysis was performed on DEGs. At the same time, in order to prevent overfitting and increase the credibility of selecting core genes, we used the least absolute shrinkage and selection operator (LASSO) Cox regression model for signature construction (28, 29). LASSO regression modeling was conducted using the R package, glmnet (30). Finally, multivariable Cox regression analyses were used for feature selection, and calculating hazard ratios (HRs) with 95% confidence intervals (CIs) (31). Genes with *p* < 0.05 was chosen for further analysis.

### Tumor Immune Infiltration Through RNA-Sequencing Expression Profiling Data

Single sample gene set enrichment analysis (ssGSEA) algorithm (https://doi.org/10.1016/j.celrep.2016.12.019, https://doi.org/10.21203/rs.3.rs-33230/v1) is scored based on 29 published immune-related genes (the immune gene set includes immune cell types, functions, and pathways) to quantify the immune infiltration level of 29 immune signatures in each HCC sample using the R package, GSVA (32), and the scores were standardized for each individual immune cell type. Besides, Tumor Immune Estimation Resource (TIMER, https://cistrome.shinyapps.io/timer/) was used to analyze the correlation between six kinds of tumor-infiltrating immune cells and selected hub genes.

### Statistical Analysis

Student's *t*-test is analyzed for comparison between two groups and one-way ANOVA is used to compare multiple groups. Survival analysis was performed using Kaplan–Meier curve by using the R packages, such as survival and survminer, and the *p*-value was calculated using the log-rank test. Chi-square test was applied to test the association of the expression of the three hub genes with clinicopathological parameters. The value of *p* < 0.05 was considered statistically significant.

## Results

### Stromal/Immune/Estimate Scores and Tumor Purity Were Significantly Associated With Prognosis and Tumor Mutation Burden

**Figure 1:**
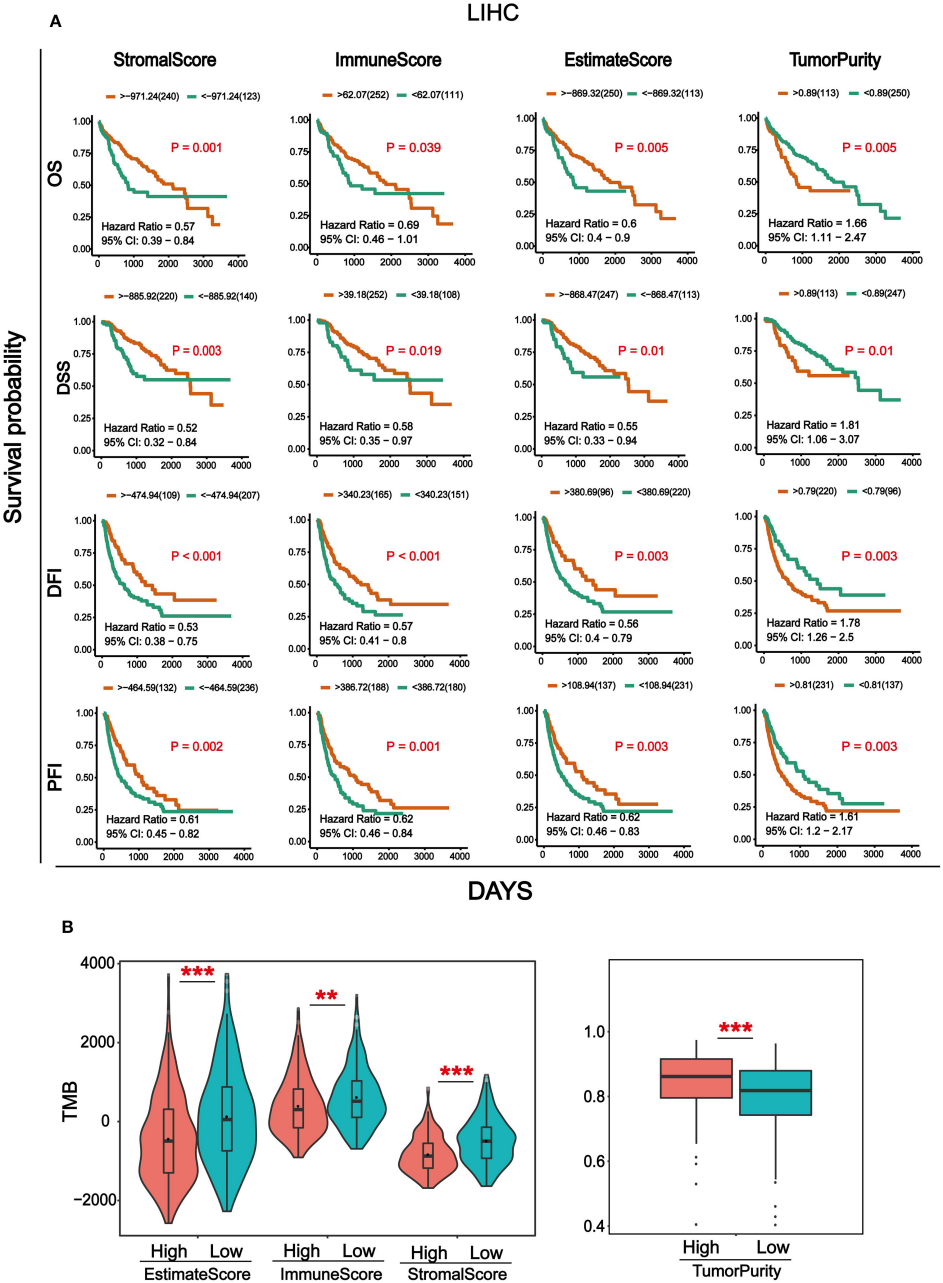
Analysis of survival and Tumor mutation burden (TMB) associated with immune/stromal/Estimate scores and tumor purity. **(A)** Kaplan–Meier survival analysis based on immune/stromal/Estimate/scores and tumor purity. Overall survival (OS), disease-specific survival (DSS), disease-free interval (DFI), progression-free interval (PFI). **(B)** The relationship of immune/stromal/Estimate/scores and tumor purity with tumor mutation burden (TMB). The samples were divided into high and low groups according to the median of score. *p < 0.05, **p < 0.01, and ***p < 0.001 between the two groups.

Transcriptome data and clinical information of HCC were downloaded from the TCGA database and integrated. Totally, 369 cancer samples were used for survival analysis (Supplementary Table 1). At the same time, in order to better understand the impact of tumor infiltrating immune and stromal cells on prognosis, we calculated the immune/stromal/Estimate scores and tumor purity based on the ESTIMATE algorithm, which helps to quantify the immune and stromal components in HCC. Sample information of overall survival (OS), disease-specific survival (DSS), disease-free interval (DFI), and progression-free interval (PFI) were collected (Supplementary Table 2). The results in Figure 1A showed that the four survival periods were significantly correlated with stromal/immune/Estimate scores and tumor purity. Elevated stromal, immune, and Estimate scores were significantly correlated with better prognosis, while higher tumor purity was significantly associated with poor patient survival rate. In addition, we also combined the clinicopathological parameters of HCC to analyze the correlation with immune score, stromal score, Estimate score, and tumor purity (Supplementary Table 3). As can be seen from Supplementary Figure 1, patients with metastasis tended to have higher immune and Estimate scores and lower tumor purity. For tumor and pathological stage, patients with increased stage tended to have high stromal, immune and Estimate score and lower tumor purity.

Tumor mutation burden (TMB) is the total number of somatic gene coding errors, base substitutions, gene insertion, or deletion errors detected per million bases (33). It is a quantitative biomarker that reflects the total number of mutations carried by tumor cells (34). In order to explore the relationship between TMB and immune/stromal/Estimate scores, tumor purity, and the mutation data of HCC from the TCGA database was downloaded for calculating the TMB. Then we divided the tumor samples into high and low groups according to the median of immune/stromal/Estimate scores and tumor purity, respectively. As can be seen from Figure 1B, TMB was significantly higher in the immune/stromal/Estimate scores in the low group, whereas it was higher in tumor purity in the high group.

### DEGs Based on Immune and Stromal Scores and Their Associated Pathways Were Identified

**Figure 2:**
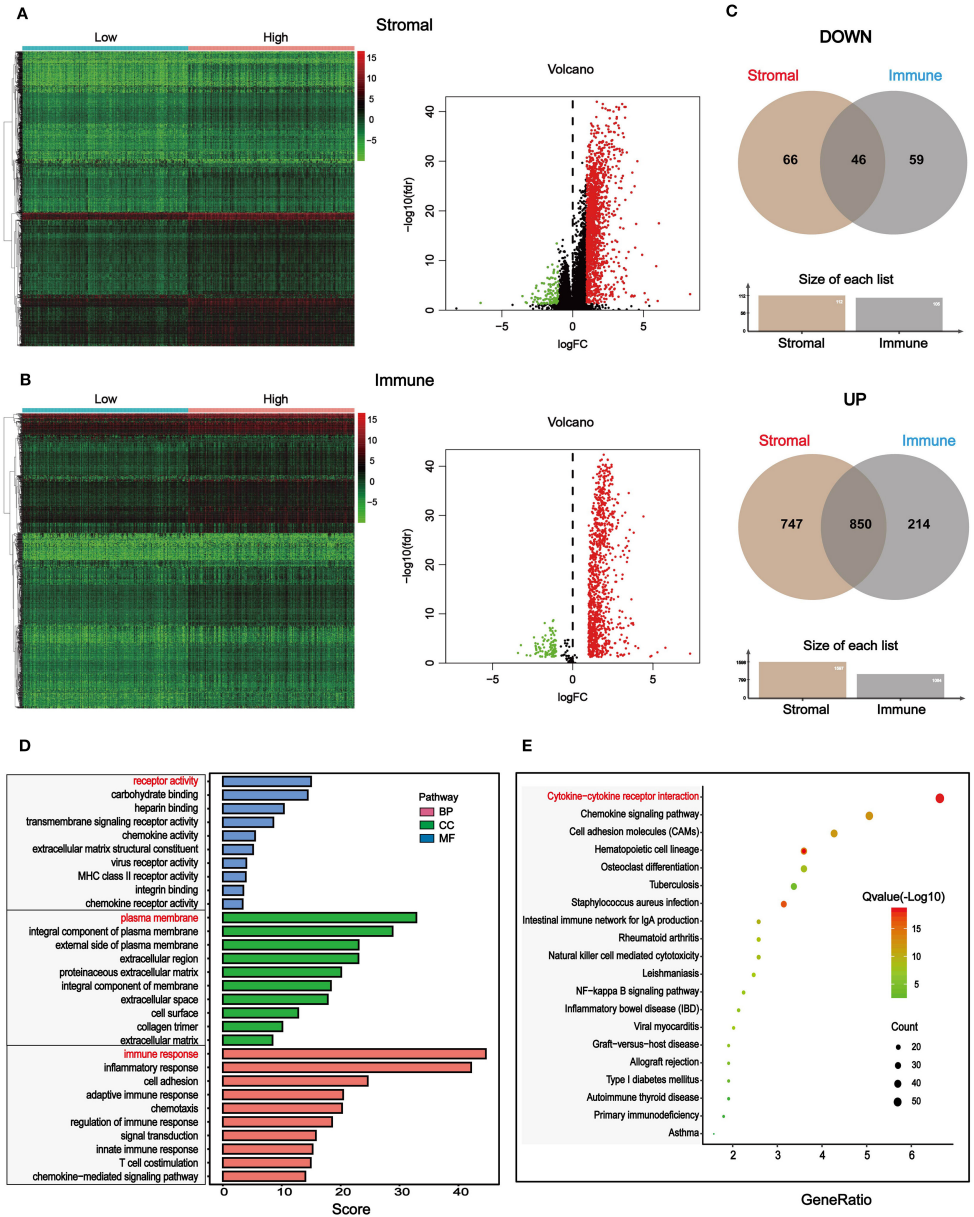
Differentially expressed genes (DEGs) based on immune and stromal scores of tumor microenvironment (TME) and their functional annotations in HCC. **(A)** Heatmaps and volcano plot of the DEGs of stromal scores (*p* < 0.05, fold change > |±1|). **(B)** Heatmaps and volcano plot of the DEGs of immune scores (*p* < 0.5, fold change > |±1|). **(C)** 46 common downregulated genes and 850 common upregulated genes of both stromal and immune scores were shown by a Venn diagram, and a total of 896 significantly different genes were obtained. **(D)** The selected DEGs were used for Gene Ontology (GO)-enrichment analysis, biological process (BP), cellular component (CC), and molecular function (MF). Top 10 GO terms were displayed, respectively. **(E)** The selected DEGs were used for Kyoto Encyclopedia of Genes and Genomes (KEGG) enrichment analysis *via* Kyoto Encyclopedia of Genes and Genomes (DAVID). Considering both the *p*-value and count number, the optimal pathway was determined.

**Figure 3:**
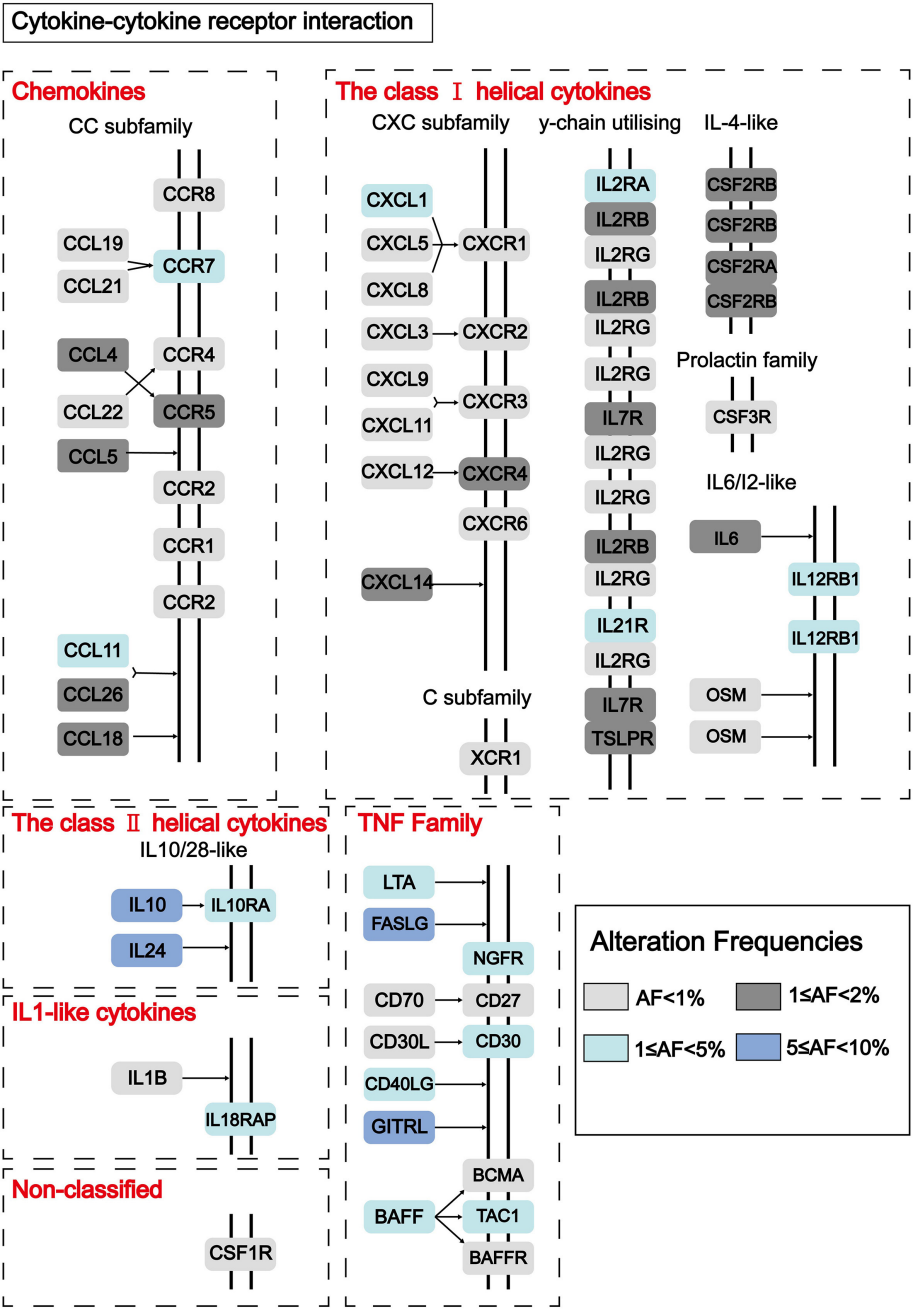
Pathway diagram showing the interaction of DEGs in the cytokine–cytokine receptor interaction pathway. Alteration frequencies of each gene were represented by the color intensity.

To find out novel genes in HCC microenvironment associated with both immune and stromal scores, we performed RNA sequencing (RNA-Seq) differential expression analysis of 374 HCC cases from TCGA cohort. First, we grouped the samples based on the median of the immune score and the stromal score, and then we conducted a difference analysis between the high and low group samples. The DEGs were displayed in the heatmap and volcano plot in Figures 2A,B. The results showed that there were 1,065 significantly upregulated genes and 105 significantly downregulated genes between high and low immune scores. Based on the difference analysis between the high and low stromal scores, 1,597 significantly upregulated genes and 112 significantly downregulated genes were obtained. By overlapping the DEGs of immune and stromal scores, we obtained a total of 896 genes (850 upregulated genes and 46 downregulated genes) for functional enrichment analysis including GO and KEGG (pathways; Figure 2C). The top 10 functional annotations of GO analysis are shown in Figure 2D. DEGs were mostly enriched in receptor activity, plasma membrane, and immune response. In addition, the enriched pathways of DEGs are displayed in Figure 2D. Considering the number of genes enriched in each pathway and the FDR value, the most important pathway was cytokine–cytokine receptor interaction (FDR = 4.24E-19). The interaction of DEGs enriched in the cytokine–cytokine receptor interaction signaling pathway is shown in Figure 3.

### Identification and Validation of Optimal Prognostic Biomarkers in HCC

**Figure 4:**
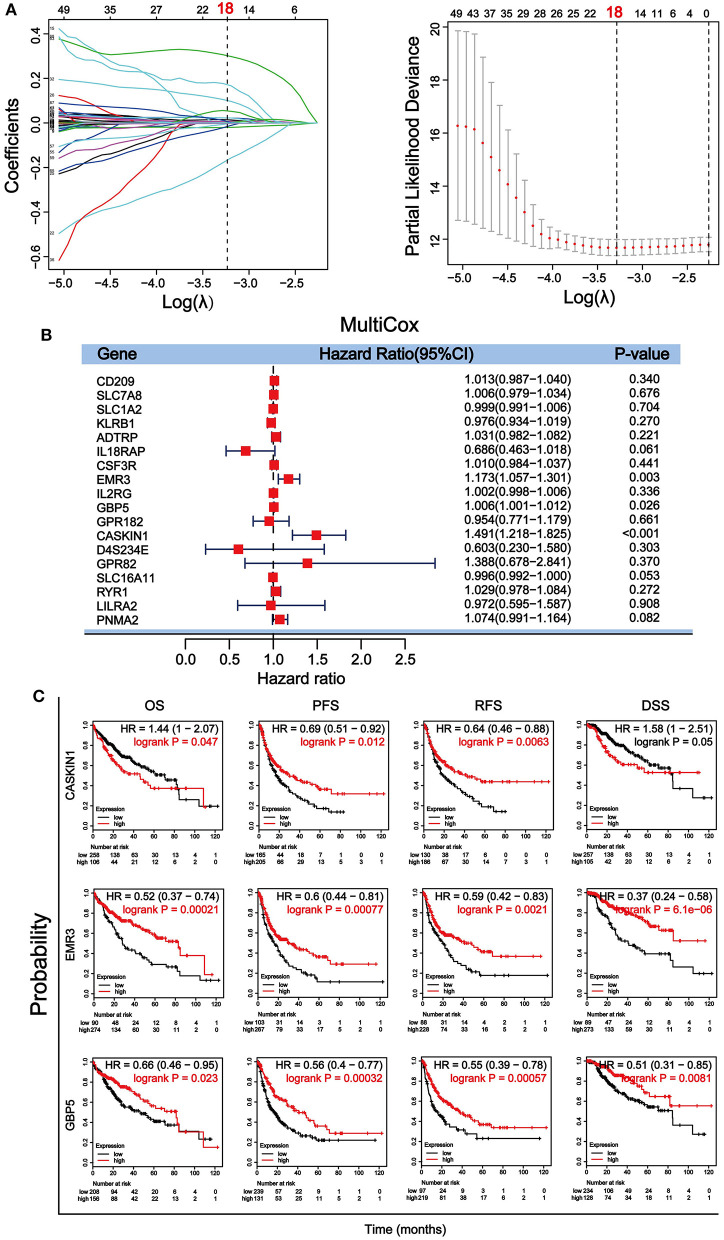
Screening and verification of prognostic genes in HCC. **(A)** Least absolute shrinkage and selection operator (LASSO) regression analysis was used to screen for genetic variables. The dotted line indicates the number of genes after screening. **(B)** Multivariate Cox regression analysis was used to further screen genes that can be used as independent prognostic factors. The value, *p* < 0.5 was considered statistically significant. **(C)** Survival verification of the three selected genes by Kaplan–Meier plotter database in HCC. Prognostic indicators include overall survival (OS), progression-free survival (PFS), relapse-free survival (RFS), disease-specific survival (DSS).

Through a well-known mathematical model, the prognostic DEGs related to the stromal–immune score that can be used as independent prognostic factors in patients with HCC was identified. We performed a univariate Cox regression analysis on the 896 DEGs related to both stromal and immune scores. Eighty-nine genes with a *p* < 0.5 were included for further analysis (Supplementary Table 4). To avoid overfitting the variables, 18 genes were screened using LASSO regression analysis (Figure 4A). Finally, three genes including guanylate binding protein 5 (GBP5), adhesion G protein-coupled receptor E3 (EMR3) and CASK interacting protein 1 (CASKIN1) were found significantly associated with HCC prognosis by multivariate Cox regression analysis (Figure 4B).

We then further verified the prognostic value of the three genes in Kaplan– Meier plotter. Survival analysis of OS, PFS, relapse-free survival (RFS), and DSS according to the expression level of the three genes were performed (Figure 4C). Our results showed that all three genes were significantly associated with the four survival parameters. High expression of the three genes predicted prolonged PFS, RFS, and DSS. High expression of EMR3 was also associated with better OS, while high expression of GBP5 was associated with better OS within 80 months and high expression of CASKIN1 was associated with worse OS before 80 months. To further validate the results, we downloaded and sorted out the GSE76427 gene expression and clinical data of a cohort of 115 HCC cases and 52 adjacent non-tumor tissue from the gene expression omnibus (GEO) database. In addition, we also collected the LIRI–JP dataset in the International Cancer Genome Consortium (ICGC) database, including 243 HCC samples and 202 adjacent samples. Similar to TCGA results, the expression of CASKIN1 gene in HCC is significantly higher than that of normal tissues, and the high expression of GBP5 gene is associated with good OS in the GEO and ICGC data analysis (Supplementary Figure 2).

### The Expression Level of GBP5, EMR3, and CASKIN1 and Their Association With Clinicopathological Parameters in HCC

**Figure 5:**
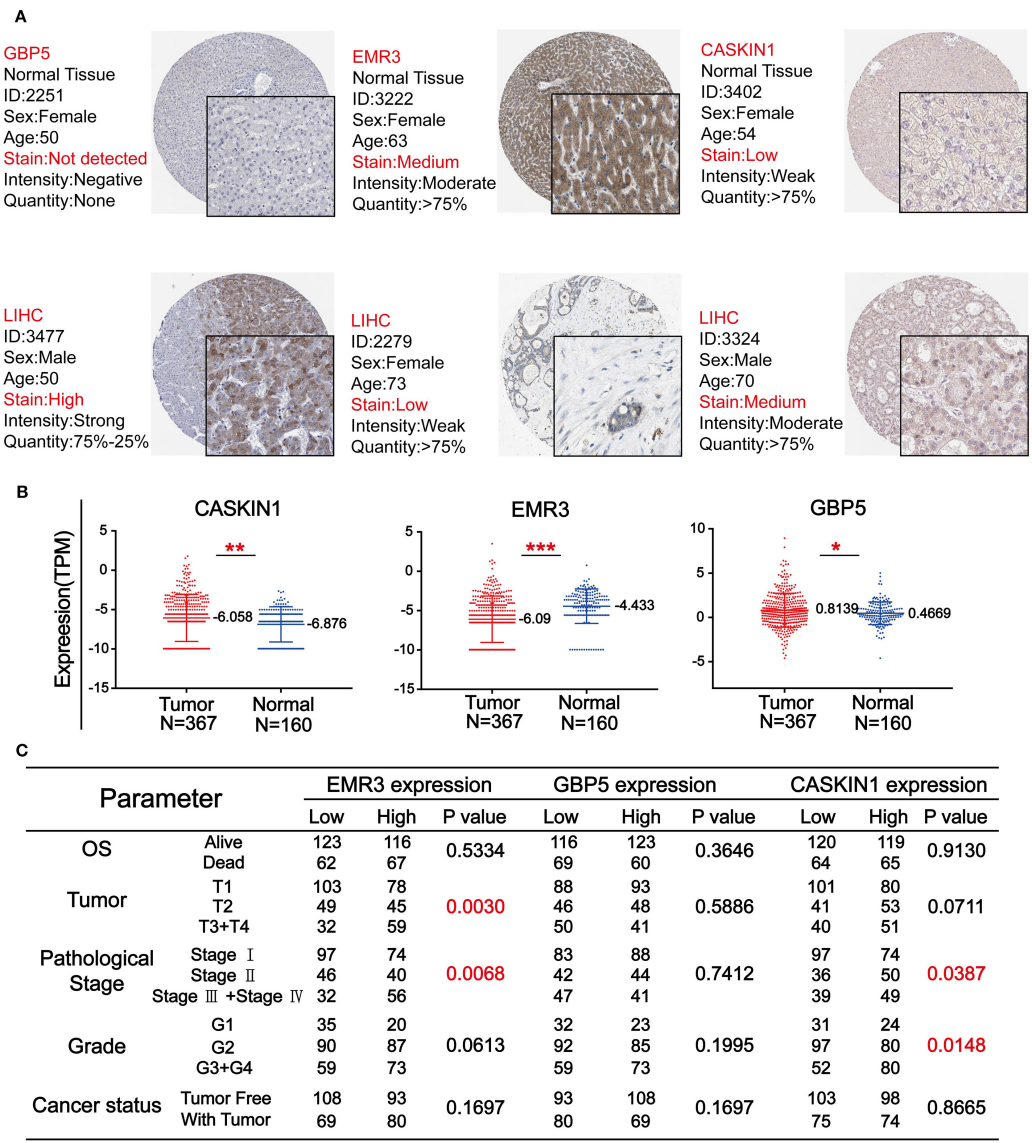
The expression level of three genes in HCC. **(A)** The immunohistochemistry (IHC) results from the Human Protein Atlas (HPA) was used to detect the protein level of three genes in normal and tumor tissues. **(B)** Comparison of the expression levels of CASKIN1, EMR3, and GBP5 genes in HCC tissues and adjacent normal tissues form the Cancer Genome Atlas (TCGA) database. The values of *p < 0.5, **p < 0.01, and ***p < 0.001 between the two groups. **(C)** Chi-square test of the clinical parameters according to the median of the expressions of the three genes.

To further confirm the importance of GBP5, EMR3, and CASKIN1 in HCC, Human Protein Atlas (HPA) database was used to compare their protein expression in normal and HCC tissues. As demonstrated in Figure 5A, GBP5 and CASKIN1 were highly expressed in HCC tissue, while EMR3 was downregulated in HCC. At the same time, we also used TCGA database to compare their expression level. CASKIN1 and GBP5 mRNA expression level was significantly increased in HCC tissue compared to adjacent normal tissues, while significantly decreased EMR3 mRNA expression was found in HCC samples compared to normal samples (Figure 5B). Moreover, we also analyzed the association of the three genes with clinicopathological parameters of HCC according to the median of their expressions (Figure 5C). Low expression of EMR3 was often found in low tumor and pathological grade and there was increased proportion of its high expression in late stage. Similar result was observed for pathological stage and tumor grade for CASKIN1 expression.

### The Association of GBP5, EMR3, and CASKIN1 Expression With Immune Cell Infiltration

**Figure 6:**
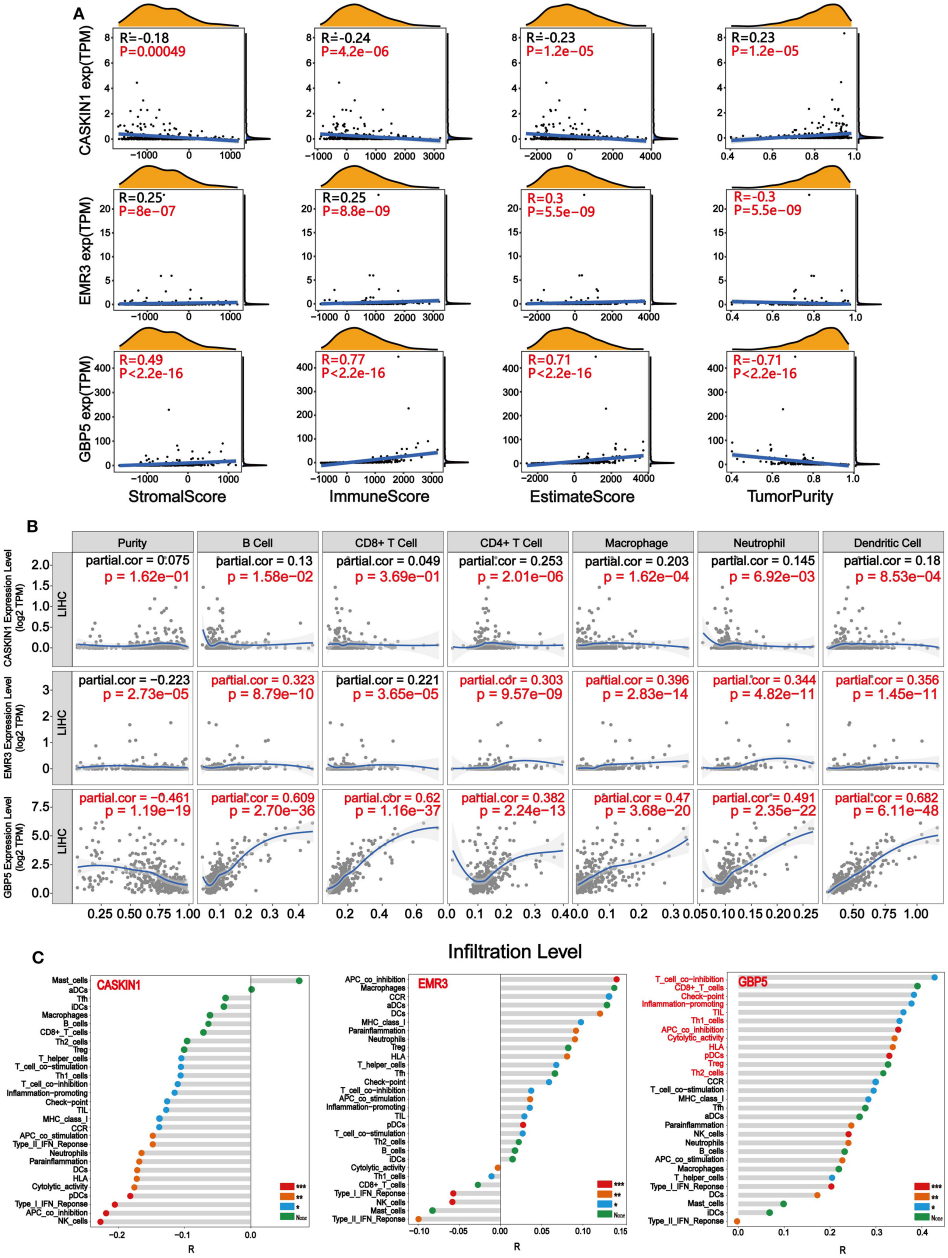
Immune infiltration related to CASKIN1, EMR3, and GBP5. **(A)** Correlation analysis between CASKIN1, EMR3, and GBP5 mRNA expression levels and immune/stromal/Estimate scores and tumor purity in hepatocellular carcinoma (HCC). **(B)** The relationship between the CASKIN1, EMR3, and GBP5 gene expression and the infiltration level of six types of immune cells in HCC *via* Tumor Immune Estimation Resource (TIMER) database. Partial Spearman's correlation and statistical analysis were performed. **(C)** Single sample gene set enrichment analysis (ssGSEA) algorithm was used to obtain the immune-infiltration levels of 29 immune cells. The correlation between the expression levels of CASKIN1, EMR3, and GBP5 expression levels and the infiltration levels of 29 immune cells was displayed using Lollipop Chart.

After identifying the prognostic value and expression level of CASKIN1, EMR3, and GBP5, we performed correlation analysis between CASKIN1, EMR3, and GBP5 expression levels and immune/stromal/Estimate scores, and tumor purity in HCC, respectively. As shown in Figure 6A, CASKIN1 expression was negatively correlated with the immune/stromal/Estimate scores and positively correlated with tumor purity. In contrast, the expression of both EMR3 and GBP5 were positively correlated with immune/stromal/Estimate scores, while negatively correlated with tumor purity. Moreover, the association of GBP5 expression with immune/stromal/Estimate scores and tumor purity was stronger than the other two genes.

To study the relationship between the expression of the three genes and immune infiltration level for HCC, scatter plots are shown with partial Spearman's correlation and statistical significance in Figure 6B. The expression of GBP5 was significantly associated with purity (*R* = −0.461). In addition, elevated EMR3 and GBP5 were significantly correlated with B cell, CD4+ T cell, macrophage, neutrophil, and dendritic cell infiltration (*p* < 0.05) and a general increase in the immune infiltration level (*R* > 0.3). It is worth noting that there is a significant positive correlation between the infiltration levels of six kinds of immune cells and the GBP5 gene expression level. Then, based on the gene expression data, we enriched the proportion of 29 immune cells in each patient with HCC through the ssGSEA algorithm, and finally obtained the infiltration level of various immune cells. The correlation of the infiltration level of the immune cells with the expression levels of three genes was calculated. As shown in Figure 6C, the CASKIN1 gene was mainly negatively correlated with immune cell infiltration and the GBP5 gene showed a positive correlation with 29 immune cells and was highly correlated with 12 immune cells (*R* > 0.3), including T-cell co-inhibition, CD8+_T cells, check-point, inflammation-promoting, TIL, Th1 cells, APC co-inhibition, cytolytic activity, HLA, pDCs, Treg, and Th2 cells. EMR3 gene showed both positive and negative correlation with 29 immune cells.

## Discussion

A large number of studies have shown that the TME plays a significant role in the occurrence, development, and metastasis of tumors (11, 13, 35). With the rapid development of bioinformatics based on tumor immunotherapy and microarray sequencing, researchers are increasingly using statistical algorithms to explore new targets for immunotherapy of HCC (19, 36, 37), including ESTIMATE algorithm, and some progress has been made (38–41).

In this study, the ESTIMATE algorithm was used to obtain the immune/stromal/Estimate scores and tumor purity of TME in (HCC). To explore the impact of the immune/stromal/Estimate scores and tumor purity on survival, we collected four types of survival data: OS, DSS, DPI, and PFI. The results showed that the differential immune/stromal/Estimate scores and tumor purity scores significantly affect survival rates. Remarkably, the high score groups of stromal/immune/Estimate scores were significantly associated with longer OS in patients with HCC among the four survival types within 7 years (Figure 1A). These findings are consistent with previous studies showing that the immune/stromal scores were significantly related to OS (40, 42). Findings in multiple cancer types revealed that TMB may play an important role in tumor immunotherapy (43–45), including bladder cancer, colorectal cancer, and non-small cell lung cancer. Therefore, we expect that the immune/stromal/Estimate scores and tumor purity in the microenvironment of HCC are related to TMB. We divided the samples into high and low groups according to the median of the TMB value to compare the correlation between the immune/stromal/Estimate scores, tumor purity, and TMB. The results showed that the higher the immune/stromal scores, the lower is the TMB value. However, the result for tumor purity was the opposite (Figure 1B). This means that the more immune/stromal cells in HCC, the harder it is to identify cancer cells (46). Previous studies have revealed that high TMB predicted worse patient outcomes than those with low TMB in patients with HCC. This is consistent with our survival results (47). In addition, we combined the immune/stromal/Estimate scores and tumor purity with the clinical parameters of HCC, such as metastasis, OS, and grade. However, there was no significant difference (Supplementary Figure 1).

Next, we grouped the samples according to the median of immune score and stromal score to find DEGs. Based on the stromal score, we got 1,579 significantly upregulated and 112 downregulated genes, and based on immune score, 1,046 significantly upregulated genes and 105 significantly downregulated genes were found (Figures 2A,B). We collected 46 DEGs that were jointly downregulated and 850 DEGs that were jointly upregulated in both immune and stromal scores for functional enrichment analysis (Figure 2C), including GO functional annotation analysis (Figure 2D) and KEGG pathway enrichment analysis (Figure 2E). The results indicated that these genes were mainly enriched in receptor activity, plasma membrane, and immune response. It can be seen from the biological process (BP), that the DEGs were closely related to immune response. Consistent with previous studies (48–50), this evidence proved that TME plays a vital role in the immunotherapy of HCC. At the same time, these genes were significantly enriched in the cytokine–cytokine receptor interaction pathway. A simplified pathway diagram in Figure 3 showed DEGs enriched in the pathway.

To evaluate the prognostic significance of these DEGs in HCC, univariate Cox regression analysis, LASSO regression analysis (Figure 4A), multivariate Cox regression analysis (Figure 4B), and Kaplan–Meier survival analysis (Figure 4C) were performed. The results demonstrated that CASKIN1, EMR3, and GBP5 were the most significant prognostic markers. Previous study has demonstrated that EMR3 is one of the adhesion G protein-coupled receptors (aGPCRs), which can be used as a modulator of immune cell function (51). It has prognostic significance in Dukes'B colon cancer (52) and glioblastoma (53). Besides, the GBP5 gene has been studied in a variety of cancers, including gastric adenocarcinoma (54), skin cutaneous melanoma (55, 56), pancreatic adenocarcinoma (57), and HCC, and GBP5 was one of the key genes in the malignant transformation induced by microcystin-LR (MC-LR) in the cell of HCC (58). In addition, GBP5 promotes immunity in mammals. It also plays an important role in regulating human macrophage pyroptosis and uniquely regulates the induction of apoptosis (59). However, the involvement of CASKIN1 gene in cancer has rarely been studied.

Besides, we tested whether these three genes are abnormally expressed in HCC. The results indicated that the expression of CASKIN1, EMR3, and GBP5 showed significant difference between the tumor and normal samples in both TCGA database and HPA (Figures 5A,B). Then, the relationship of the expression of the three genes and their clinical parameters were analyzed *via* the Chi-square test (Figure 5C). Among them, the expression of EMR3 showed significant difference in the clinical parameters of tumor and pathological stage. There was also significant difference for CASKIN1 in the pathological stage and grade. These results indicated that CASKIN1 and EMR3 were possibly involved in the progression of HCC. At the same time, we also verified the significance of the three prognostic genes in the GEO and ICGC database (Supplementary Figure 2).

Finally, to further analyze the significance of the three genes in tumor immune infiltration, we analyzed the correlation between the three genes and the immune/stromal/Estimate scores and tumor purity, respectively (Figure 6A). At the same time, the TIMER database was applied to assess the correlation between the expression of the three genes and the infiltration scores of six immune cell types. In both the analyses, the GBP5 gene showed a strong correlation with the degree of immune infiltration (Figure 6B). Next, the ssGSEA algorithm was used to evaluate RNA-Seq expression profile data to detect the infiltration of immune cells in tumor tissues of HCC. The correlation analysis between the expression of CASKIN1, EMR3, and GBP5 genes and the immune infiltration scores of 29 immune cell types was performed (Figure 6C). The results showed that GBP5 gene expression exhibited strong positive correlation with 12 kinds of immune cells (*R* > 0.3, *p* < 0.5), which verified the results in Figures 6A,B, suggesting that GBP5 may be an important target for targeted immunotherapy of HCC.

In summary, our study identified three TME-related prognostic markers in HCC. CASKIN1 was overexpressed in tumor and its high expression was associated with poor OS, while high expression of EMR3 and GBP5 were associated with better survival. However, the prognostic value of the three genes warrants further validation by more clinical data. Importantly, the GBP5 gene was highly expressed in HCC and strongly correlated with immune cell infiltration. It holds a great potential as a candidate for targeted immunotherapy of HCC.

## Data Availability Statement

RNA sequencing and clinicopathological data of HCC patients were downloaded from GDC database (https://portal.gdc.cancer.gov/) - https://gdc-hub.s3.us-east-1.amazonaws.com/latest/TCGA-LIHC.htseq_fpkm.tsv.gz.

## Author Contributions

SX, JL, and JS wrote the paper. YZha, XW, XY, and ML edited the manuscript. PK, CC, FD, QW, and YZhe prepared and adjusted the figures. TY and ZX designed the study, provided funding, and reviewed the manuscript.

## Conflict of Interest

The authors declare that the research was conducted in the absence of any commercial or financial relationships that could be construed as a potential conflict of interest.

## Supplementary Material

Supplementary Figure 1 Analysis of clinicopathological indicators associated with immune/stromal/Estimate scores and tumor purity. Clinical parameters include metastasis (M0, M1), overall survival (OS) (alive, dead), grade (G1, G2, G3, and G4), tumor (T1, T2, T3, and T4) and pathological stage (Stage I; Stage II, Stage III, Stage IV). Student's *t*-test was used for comparison between the two groups of data, and a one-way ANOVA was applied to compare multiple groups.

Supplementary Figure 2 Validation of prognostic genes in the gene expression omnibus (GEO) and the International Cancer Genome Consortium (ICGC) database. **(A)** The expression levels and verification of CASKIN1, EMR3, and GBP5 genes in HCC tissues and adjacent normal tissues from GSE76427 dataset in the GEO database. **p* < 0.05, ***p* < 0.01, and ****p* < 0.001 between the two groups. **(B)** Survival verification of the three prognostic genes by gene expression omnibus (GEO) database in hepatocellular carcinoma (HCC) (*p* < 0.05 in Log-rank test). **(C)** The expression levels and verification of CASKIN1, EMR3, and GBP5 genes in HCC samples and adjacent samples from LIRI-JP dataset in the ICGC database. **p* < 0.05, ***p* < 0.01, and ****p* < 0.001 between the two groups. **(D)** Survival validation of the three prognostic genes using data from ICGC database in HCC (*p* < 0.05 in Log-rank test).

Supplementary Table 1 The immune/stromal/Estimate scores and tumor purity of HCC were calculated by using ESTIMATE algorithm.

Supplementary Table 2 The sample information of overall survival (OS), disease-specific survival (DSS), disease-free interval (DFI), and progression-free interval (PFI).

Supplementary Table 3 The immune/stromal/Estimate scores and tumor purity with the clinical parameters of hepatocellular carcinoma (HCC), including tumor, metastasis, OS, grade, stage, and cancer status.

Supplementary Table 4 The results of univariate COX regression analysis.

## References

[1] Fu Q Yang F Xiang T Huai G Yang X Wei L. Author correction: a novel microRNA signature predicts survival in liver hepatocellular carcinoma after hepatectomy. Sci Rep. (2018) 8:9395. 10.1038/s41598-018-27641-5 29904077 PMC6002540

[2] Farazi PA DePinho RA. Hepatocellular carcinoma pathogenesis: from genes to environment. Nat Rev Cancer. (2006) 6:674–87. 10.1038/nrc1934 16929323

[3] Llovet JM Montal R Villanueva A. Randomized trials and endpoints in advanced HCC: role of PFS as a surrogate of survival. J Hepatol. (2019) 70:1262–77. 10.1016/j.jhep.2019.01.028 30943423 PMC12452112

[4] DeSantis CE Ma J Gaudet MM Newman LA Miller KD Goding Sauer A. Breast cancer statistics, 2019. CA Cancer J Clin. (2019) 69:438–51. 10.3322/caac.21583 31577379

[5] Global Burden of Disease Cancer Collaboration, Fitzmaurice C Abate D Abbasi N Abbastabar H Abd-Allah F. Global, regional, and national cancer incidence, mortality, years of life lost, years lived with disability, and disability-adjusted life-years for 29 cancer groups, 1990 to 2017: a systematic analysis for the Global Burden of Disease Study. JAMA Oncol. (2019) 5:1749–68. 10.1001/jamaoncol.2019.2996 31560378 PMC6777271

[6] Fu Y Liu S Zeng S Shen H. From bench to bed: the tumor immune microenvironment and current immunotherapeutic strategies for hepatocellular carcinoma. J Exp Clin Cancer Res. (2019) 38:396. 10.1186/s13046-019-1396-4 31500650 PMC6734524

[7] Hato T Goyal L Greten TF Duda DG Zhu AX. Immune checkpoint blockade in hepatocellular carcinoma: current progress and future directions. Hepatology. (2014) 60:1776–82. 10.1002/hep.27246 24912948 PMC4211962

[8] Zhang HH Mei MH Fei R Liu F Wang JH Liao WJ. Regulatory T cells in chronic hepatitis B patients affect the immunopathogenesis of hepatocellular carcinoma by suppressing the anti-tumour immune responses. J Viral Hepat. (2010) 17(Suppl. 1):34–43. 10.1111/j.1365-2893.2010.01269.x 20586932

[9] Xu WH Xu Y Wang J Wan FN Wang HK Cao DL. Prognostic value and immune infiltration of novel signatures in clear cell renal cell carcinoma microenvironment. Aging. (2019) 11:6999–7020. 10.18632/aging.102233 31493764 PMC6756904

[10] Jia D Li S Li D Xue H Yang D Liu Y. Mining TCGA database for genes of prognostic value in glioblastoma microenvironment. Aging. (2018) 10:592–605. 10.18632/aging.101415 29676997 PMC5940130

[11] Quail DF Joyce AJ. Microenvironmental regulation of tumor progression and metastasis. Nat Med. (2013) 19:1423–37. 10.1038/nm.3394 24202395 PMC3954707

[12] Li X Wenes M Romero P Huang SC Fendt SM Ho PC. Navigating metabolic pathways to enhance antitumour immunity and immunotherapy. Nat Rev Clin Oncol. (2019) 16:425–41. 10.1038/s41571-019-0203-7 30914826

[13] Belli C Trapani D Viale G D'Amico P Duso BA Della Vigna P. Targeting the microenvironment in solid tumors. Cancer Treat Rev. (2018) 65:22–32. 10.1016/j.ctrv.2018.02.004 29502037

[14] Eggert T Greten FT. Tumor regulation of the tissue environment in the liver. Pharmacol Ther. (2017) 173:47–57. 10.1016/j.pharmthera.2017.02.005 28167218 PMC5408316

[15] Yang S Gao H. Nanoparticles for modulating tumor microenvironment to improve drug delivery and tumor therapy. Pharmacol Res. (2017) 126:97–108. 10.1016/j.phrs.2017.05.004 28501517

[16] Zhou L Huang W Yu HF Feng YJ Teng X. Exploring TCGA database for identification of potential prognostic genes in stomach adenocarcinoma. Cancer Cell Int. (2020) 20:264. 10.1186/s12935-020-01351-3 32581654 PMC7310509

[17] Oliver GR Hart SN Klee EW. Bioinformatics for clinical next generation sequencing. Clin Chem. (2015) 61:124–35. 10.1373/clinchem.2014.224360 25451870

[18] Yin Z Lan H Tan G Lu M Vasilakos AV Liu W. Computing platforms for big biological data analytics: perspectives and challenges. Comput Struct Biotechnol J. (2017) 15:403–11. 10.1016/j.csbj.2017.07.004 28883909 PMC5581845

[19] Hohenberger P. Locoregional recurrence of rectal cancer: biological and technical aspects of surgical failure. Recent Results Cancer Res. (1998) 146:127–40. 10.1007/978-3-642-71967-7_12 9670256

[20] Olsen LR Campos B Barnkob MS Winther O Brusic V Andersen MH. Bioinformatics for cancer immunotherapy target discovery. Cancer Immunol Immunother. (2014) 63:1235–49. 10.1007/s00262-014-1627-7 25344903 PMC11029190

[21] He KY Ge D He MM. Big data analytics for genomic medicine. Int J Mol Sci. (2017) 18:412. 10.3390/ijms18020412 28212287 PMC5343946

[22] Wu J Xu WH Wei Y Qu YY Zhang HL Ye DW. An integrated score and nomogram combining clinical and immunohistochemistry factors to predict high ISUP grade clear cell renal cell carcinoma. Front Oncol. (2018) 8:634. 10.3389/fonc.2018.00634 30619768 PMC6305456

[23] Yoshihara K Shahmoradgoli M Martinez E Vegesna R Kim H Torres-Garcia W. Inferring tumour purity and stromal and immune cell admixture from expression data. Nat Commun. (2013) 4:2612. 10.1038/ncomms3612 24113773 PMC3826632

[24] Alonso MH Ausso S Lopez-Doriga A Cordero D Guino E Sole X. Comprehensive analysis of copy number aberrations in microsatellite stable colon cancer in view of stromal component. Br J Cancer. (2017) 117:421–31. 10.1038/bjc.2017.208 28683472 PMC5537504

[25] Hutter C Zenklusen JC. The cancer genome atlas: creating lasting value beyond its data. Cell. (2018) 173:283–5. 10.1016/j.cell.2018.03.042 29625045

[26] Wang Z Jensen MA Zenklusen JC. A practical guide to The Cancer Genome Atlas (TCGA). Methods Mol Biol. (2016) 1418:111–41. 10.1007/978-1-4939-3578-9_6 27008012

[27] Huang da W Sherman BT Lempicki RA. Systematic and integrative analysis of large gene lists using DAVID bioinformatics resources. Nat Protoc. (2009) 4:44–57. 10.1038/nprot.2008.211 19131956

[28] Lao J Chen Y Li ZC Li Q Zhang J Liu J. A deep learning-based radiomics model for prediction of survival in glioblastoma multiforme. Sci Rep. (2017) 7:10353. 10.1038/s41598-017-10649-8 28871110 PMC5583361

[29] Li J Liu C Chen Y Gao C Wang M Ma X. Tumor characterization in breast cancer identifies immune-relevant gene signatures associated with prognosis. Front Genet. (2019) 10:1119. 10.3389/fgene.2019.01119 31781173 PMC6861325

[30] Friedman J Hastie T Tibshirani R. Regularization paths for generalized linear models via coordinate descent. J Stat Softw. (2010) 33:1–22. 10.18637/jss.v033.i01 20808728 PMC2929880

[31] Bolzetta F Wetle T Besdine R Noale M Cester A Crepaldi G. The relationship between different settings of medical service and incident frailty. Exp Gerontol. (2018) 108:209–14. 10.1016/j.exger.2018.04.023 29730329 PMC6326371

[32] Hanzelmann S Castelo R Guinney J. GSVA: gene set variation analysis for microarray and RNA-seq data. BMC Bioinformatics. (2013) 14:7. 10.1186/1471-2105-14-7 23323831 PMC3618321

[33] Chan TA Yarchoan M Jaffee E Swanton C Quezada SA Stenzinger A. Development of tumor mutation burden as an immunotherapy biomarker: utility for the oncology clinic. Ann Oncol. (2019) 30:44–56. 10.1093/annonc/mdy495 30395155 PMC6336005

[34] Fancello L Gandini S Pelicci PG Mazzarella L. Tumor mutational burden quantification from targeted gene panels: major advancements and challenges. J Immunother Cancer. (2019) 7:183. 10.1186/s40425-019-0647-4 31307554 PMC6631597

[35] Laplane L Duluc D Bikfalvi A Larmonier N Pradeu T. Beyond the tumour microenvironment. Int J Cancer. (2019) 145:2611–2618. 10.1002/ijc.32343 30989643 PMC6766895

[36] Deng F Chen D Wei X Lu S Luo X He J. Development and validation of a prognostic classifier based on HIF-1 signaling for hepatocellular carcinoma. Aging. (2020) 12:3431–50. 10.18632/aging.102820 32084009 PMC7066907

[37] Kiprenskii Iu V Erofeev GP. [The immunogenicity of different organs and the characteristics of immunosuppressive therapy during their allotransplantation]. Khirurgiia. (1992) 3:123–32. 1434354

[38] Deng Z Wang J Xu B Jin Z Wu G Zeng. Mining TCGA database for tumor microenvironment-related genes of prognostic value in hepatocellular carcinoma. Biomed Res Int. (2019) 2019:2408348. 10.1155/2019/2408348 31828095 PMC6885833

[39] Hsiao YW Chiu LT Chen CH Shih WL Lu TP. Tumor-infiltrating leukocyte composition and prognostic power in hepatitis B- and hepatitis C-related hepatocellular carcinomas. Genes. (2019) 10:630. 10.3390/genes10080630 31434354 PMC6722571

[40] Pan L Fang J Chen MY Zhai ST Zhang B Jiang ZY. Promising key genes associated with tumor microenvironments and prognosis of hepatocellular carcinoma. World J Gastroenterol. (2020) 26:789–803. 10.3748/wjg.v26.i8.789 32148377 PMC7052538

[41] Zanjani HS Mariani J Herrup K. Cell loss in the inferior olive of the staggerer mutant mouse is an indirect effect of the gene. J Neurogenet. (1990) 6:229–41. 10.3109/01677069009107113 2231177

[42] Tian Z Wang Z Chen Y Qu S Liu C Chen F. Bioinformatics analysis of prognostic tumor microenvironment-related genes in the tumor microenvironment of hepatocellular carcinoma. Med Sci Monit. (2020) 26:e922159. 10.12659/MSM.922159 32231177 PMC7146066

[43] Rizvi NA Hellmann MD Snyder A Kvistborg P Makarov V Havel JJ. Cancer immunology. Mutational landscape determines sensitivity to PD-1 blockade in non-small cell lung cancer. Science. (2015) 348:124–8. 10.1126/science.aaa1348 25765070 PMC4993154

[44] Samstein RM Lee CH Shoushtari AN Hellmann MD Shen R Janjigian YY. Tumor mutational load predicts survival after immunotherapy across multiple cancer types. Nat Genet. (2019) 51:202–06. 10.1038/s41588-018-0312-8 30643254 PMC6365097

[45] Snyder A Wolchok JD Chan TA. Genetic basis for clinical response to CTLA-4 blockade. N Engl J Med. (2015) 372:783. 10.1056/NEJMc1415938 25693024

[46] Gandara DR Paul SM Kowanetz M Schleifman E Zou W Li Y. Blood-based tumor mutational burden as a predictor of clinical benefit in non-small-cell lung cancer patients treated with atezolizumab. Nat Med. (2018) 24:1441–8. 10.1038/s41591-018-0134-3 30082870

[47] Shrestha R Prithviraj P Anaka M Bridle KR Crawford DHG Dhungel B. Monitoring immune checkpoint regulators as predictive biomarkers in hepatocellular carcinoma. Front Oncol. (2018) 8:269. 10.3389/fonc.2018.00269 30057891 PMC6053505

[48] Pineiro Fernandez J Luddy KA Harmon C O'Farrelly C. Hepatic tumor microenvironments and effects on NK cell phenotype and function. Int J Mol Sci. (2019) 20:4131. 10.3390/ijms20174131 31450598 PMC6747260

[49] Wu Q Zhou L Lv D Zhu X Tang H. Exosome-mediated communication in the tumor microenvironment contributes to hepatocellular carcinoma development and progression. J Hematol Oncol. (2019) 12:53. 10.1186/s13045-019-0739-0 31142326 PMC6542024

[50] Zhang FP Huang YP Luo WX Deng WY Liu CQ Xu LB. Construction of a risk score prognosis model based on hepatocellular carcinoma microenvironment. World J Gastroenterol. (2020) 26:134–53. 10.3748/wjg.v26.i2.134 31969776 PMC6962430

[51] Hamann J Hsiao CC Lee CS Ravichandran KS Lin HH. Adhesion GPCRs as modulators of immune cell function. Handb Exp Pharmacol. (2016) 234:329–50. 10.1007/978-3-319-41523-9_15 27832495

[52] Wang Y Jatkoe T Zhang Y Mutch MG Talantov D Jiang J. Gene expression profiles and molecular markers to predict recurrence of Dukes' B colon cancer. J Clin Oncol. (2004) 22:1564–71. 10.1200/JCO.2004.08.186 15051756

[53] Kane AJ Sughrue ME Rutkowski MJ Phillips JJ Parsa AT. EMR-3: a potential mediator of invasive phenotypic variation in glioblastoma and novel therapeutic target. Neuroreport. (2010) 21:1018–22. 10.1097/WNR.0b013e32833f19f2 20827226 PMC3064464

[54] Patil PA Blakely AM Lombardo KA Machan JT Miner TJ Wang LJ. Expression of PD-L1, indoleamine 2,3-dioxygenase and the immune microenvironment in gastric adenocarcinoma. Histopathology. (2018) 73:124–36. 10.1111/his.13504 29489025

[55] Chen W Cheng P Jiang J Ren Y Wu D Xue D. Epigenomic and genomic analysis of transcriptome modulation in skin cutaneous melanoma. Aging. (2020) 12:12703–25. 10.18632/aging.103115 32639949 PMC7377867

[56] Wang Q Wang X Liang Q Wang S Xiwen L Pan F. Distinct prognostic value of mRNA expression of guanylate-binding protein genes in skin cutaneous melanoma. Oncol Lett. (2018) 15:7914–2. 10.3892/ol.2018.8306 29725478 PMC5920493

[57] He QL Jiang HX Zhang XL Qin SY. Relationship between a 7-mRNA signature of the pancreatic adenocarcinoma microenvironment and patient prognosis (a STROBE-compliant article). Medicine. (2020) 99:e21287. 10.1097/MD.0000000000021287 32702921 PMC7373597

[58] Chen HQ Zhao J Li Y He LX Huang YJ Shu WQ. Gene expression network regulated by DNA methylation and microRNA during microcystin-leucine arginine induced malignant transformation in human hepatocyte L02 cells. Toxicol Lett. (2018) 289:42–53. 10.1016/j.toxlet.2018.03.003 29518473

[59] Fisch D Bando H Clough B Hornung V Yamamoto M Shenoy AR. Human GBP1 is a microbe-specific gatekeeper of macrophage apoptosis and pyroptosis. EMBO J. (2019) 38:e100926. 10.15252/embj.2018100926 31268602 PMC6600649

