# Supplementary figures and images for: Identification of Prognostic Genes in the Tumor Microenvironment of Hepatocellular Carcinoma
Source: Front Immunol. 2021 Apr 7;12:653836. doi: 10.3389/fimmu.2021.653836 (PMC8059369; doi:10.3389/fimmu.2021.653836)

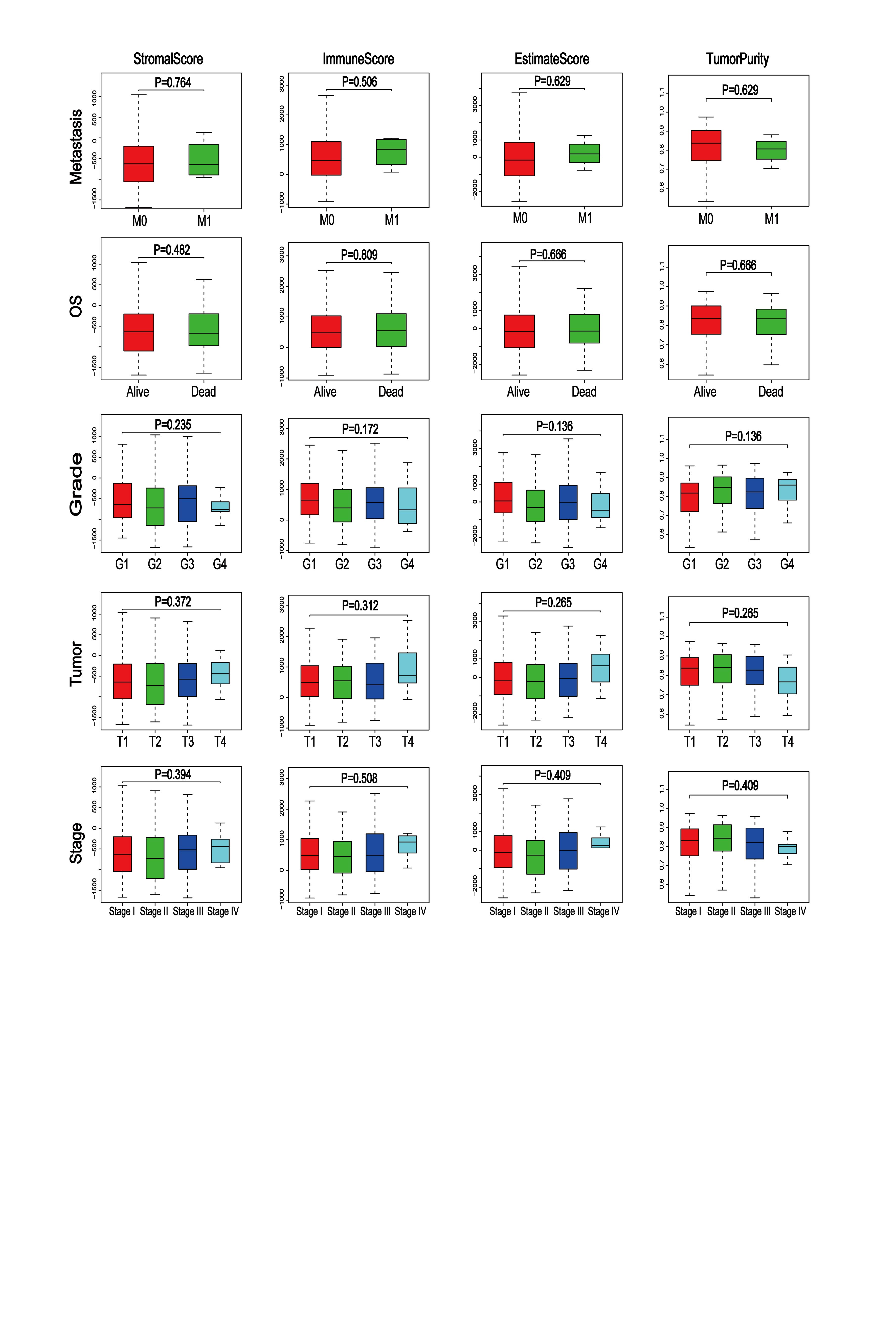

Supplement: Supplementary Figure 1 — Analysis of clinicopathological indicators associated with immune/stromal/Estimate scores and tumor purity. Clinical parameters include metastasis (M0, M1), overall survival (OS) (alive, dead), grade (G1, G2, G3, and G4), tumor (T1, T2, T3, and T4) and pathological stage (Stage I; Stage II, Stage III, Stage IV). Student's t-test was used for comparison between the two groups of data, and a one-way ANOVA was applied to compare multiple groups. [file Image_1.JPEG]

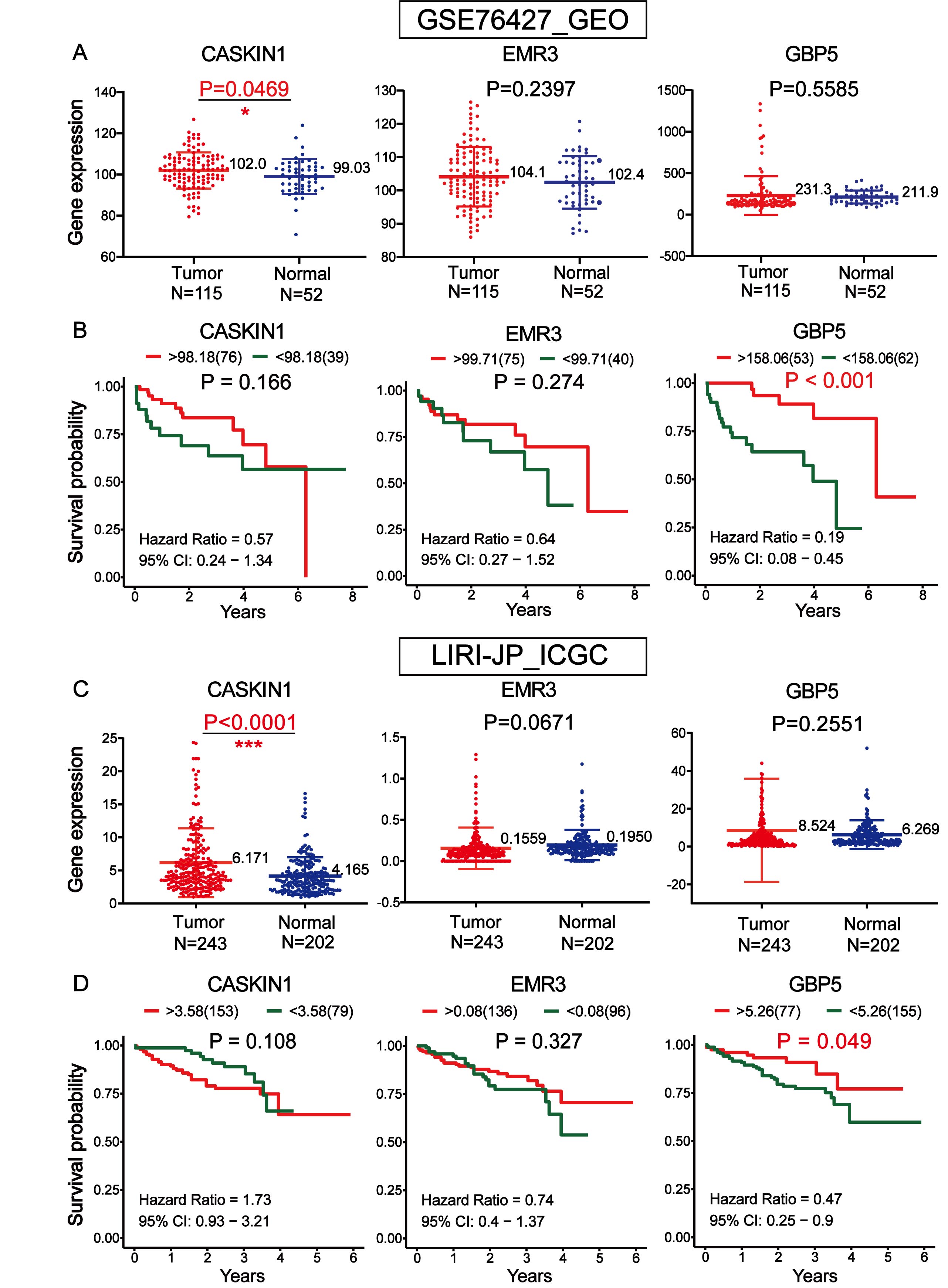

Supplement: Supplementary Figure 2 — Validation of prognostic genes in the gene expression omnibus (GEO) and the International Cancer Genome Consortium (ICGC) database. (A) The expression levels and verification of CASKIN1, EMR3, and GBP5 genes in HCC tissues and adjacent normal tissues from GSE76427 dataset in the GEO database. *p < 0.05, **p < 0.01, and ***p < 0.001 between the two groups. (B) Survival verification of the three prognostic genes by gene expression omnibus (GEO) database in hepatocellular carcinoma (HCC) (p < 0.05 in Log-rank test). (C) The expression levels and verification of CASKIN1, EMR3, and GBP5 genes in HCC samples and adjacent samples from LIRI-JP dataset in the ICGC database. *p < 0.05, **p < 0.01, and ***p < 0.001 between the two groups. (D) Survival validation of the three prognostic genes using data from ICGC database in HCC (p < 0.05 in Log-rank test). [file Image_2.jpg]
